# Environment-insensitive and gate-controllable photocurrent enabled by bandgap engineering of MoS_2_ junctions

**DOI:** 10.1038/srep44768

**Published:** 2017-03-21

**Authors:** Fu-Yu Shih, Yueh-Chun Wu, Yi-Siang Shih, Ming-Chiuan Shih, Tsuei-Shin Wu, Po-Hsun Ho, Chun-Wei Chen, Yang-Fang Chen, Ya-Ping Chiu, Wei-Hua Wang

**Affiliations:** 1 Department of Physics, National Taiwan University, Taipei 106, Taiwan; 2 Institute of Atomic and Molecular Sciences, Academia Sinica, Taipei 106, Taiwan; 3 Department of Physics, National Sun Yat-sen University, Kaohsiung, Taiwan; 4 Department of Materials Science and Engineering, National Taiwan University, Taipei 106, Taiwan; 5 Institute of Physics, Academia Sinica, Taipei 115, Taiwan

## Abstract

Two-dimensional (2D) materials are composed of atomically thin crystals with an enormous surface-to-volume ratio, and their physical properties can be easily subjected to the change of the chemical environment. Encapsulation with other layered materials, such as hexagonal boron nitride, is a common practice; however, this approach often requires inextricable fabrication processes. Alternatively, it is intriguing to explore methods to control transport properties in the circumstance of no encapsulated layer. This is very challenging because of the ubiquitous presence of adsorbents, which can lead to charged-impurity scattering sites, charge traps, and recombination centers. Here, we show that the short-circuit photocurrent originated from the built-in electric field at the MoS_2_ junction is surprisingly insensitive to the gaseous environment over the range from a vacuum of 1 × 10^−6^  Torr to ambient condition. The environmental insensitivity of the short-circuit photocurrent is attributed to the characteristic of the diffusion current that is associated with the gradient of carrier density. Conversely, the photocurrent with bias exhibits typical persistent photoconductivity and greatly depends on the gaseous environment. The observation of environment-insensitive short-circuit photocurrent demonstrates an alternative method to design device structure for 2D-material-based optoelectronic applications.

Generally, two-dimensional (2D) materials, composed of atomically thin crystals, exhibit an enormous surface-to-volume ratio, and the physical properties of 2D materials, including electrical, optical, and mechanical properties, are easily subjected to the change of the chemical environment^1^,^2^,^3^,^4^,^5^,^6^. Regarding the electrical property, the carrier transport in 2D materials is very sensitive to the presence of extrinsic adsorbents, which typically cause charged-impurity scattering, charge trapping, and recombination centers^7^,^8^,^9^,^10^,^11^, leading to degradation of the transport characteristics^12^,^13^,^14^. Although various encapsulation methods have been developed^15^,^16^,^17^,^18^,^19^, it is intriguing to explore methods to control the transport properties in the circumstance of no encapsulated layer. Here, we demonstrated that the short-circuit photocurrent enabled by the built-in electric field at the MoS_2_ junction is surprisingly insensitive to the gaseous environment, which is very uncommon in the photoresponse of thin transition-metal dichalcogenides (TMDCs). We exploit the unique property of 2D TMDCs, in which the electronic band structures are associated with the number of the layered materials^20^,^21^, to create a junction structure. For molybdenum disulfide (MoS_2_), the transition changes from direct bandgap (1.9 eV) in monolayer MoS_2_ to indirect bandgap (1.3 eV) in bulk MoS_2_^22^,^23^,^24^. This layer-dependent electronic structure therefore offers a distinct approach for designing the MoS_2_ junction based on homogeneous material of the TMDCs^25^,^26^,^27^,^28^.

In this work, we fabricated atomic thin MoS_2_ junction phototransistors with different MoS_2_ layers and studied their photoresponse behavior at different source-drain bias and gaseous environment. The difference of band gap in different thickness of few layer MoS_2_ was utilized to create a built-in electric field. Interestingly, we observed that the short-circuit photocurrent due to the photovoltaic (PV) effect^29^ is insensitive to the gaseous environment over the range from a vacuum of 1 × 10^−6^ Torr to ambient condition. This environmental insensitivity can be well ascribed to the unique characteristic of diffusion current that is associated with the carrier density gradient. Conversely, the photocurrent under bias exhibits persistent photoconductivity (PPC) and highly depends on the gaseous environment. The scanning tunneling microscopy and spectroscopy (STM and STS) measurements reveal the energy profile at the MoS_2_ junction, confirming the presence of the band offset. Moreover, we show that the MoS_2_ junction phototransistors exhibit gate-voltage tunable open-circuit voltage and short-circuit photocurrent, demonstrating the capability for regulating the current-voltage characteristics of the MoS_2_ junction via electric field effect.

The details of sample fabrication processes are described in the Supporting Information S1. In brief, MoS_2_ junctions with different layers were prepared by mechanically exfoliating MoS_2_ crystals onto SiO_2_(300 nm)/Si substrates. We then identify MoS_2_ flakes with different numbers of layers using optical microscopy. The thicknesses of the flakes of MoS_2_ were characterized using atomic force microscopy and Raman spectroscopy (Supporting Information S2). We then employ a resist-free approach to define the electrical contact to the MoS_2_ junction to avoid the resist residue which may cause extra carrier scattering. We then deposited Au (50 nm) for the electrical contacts by e-beam evaporation at a base pressure of 1 × 10^−7^ Torr.

## Results and Discussion

**Figure 1:**
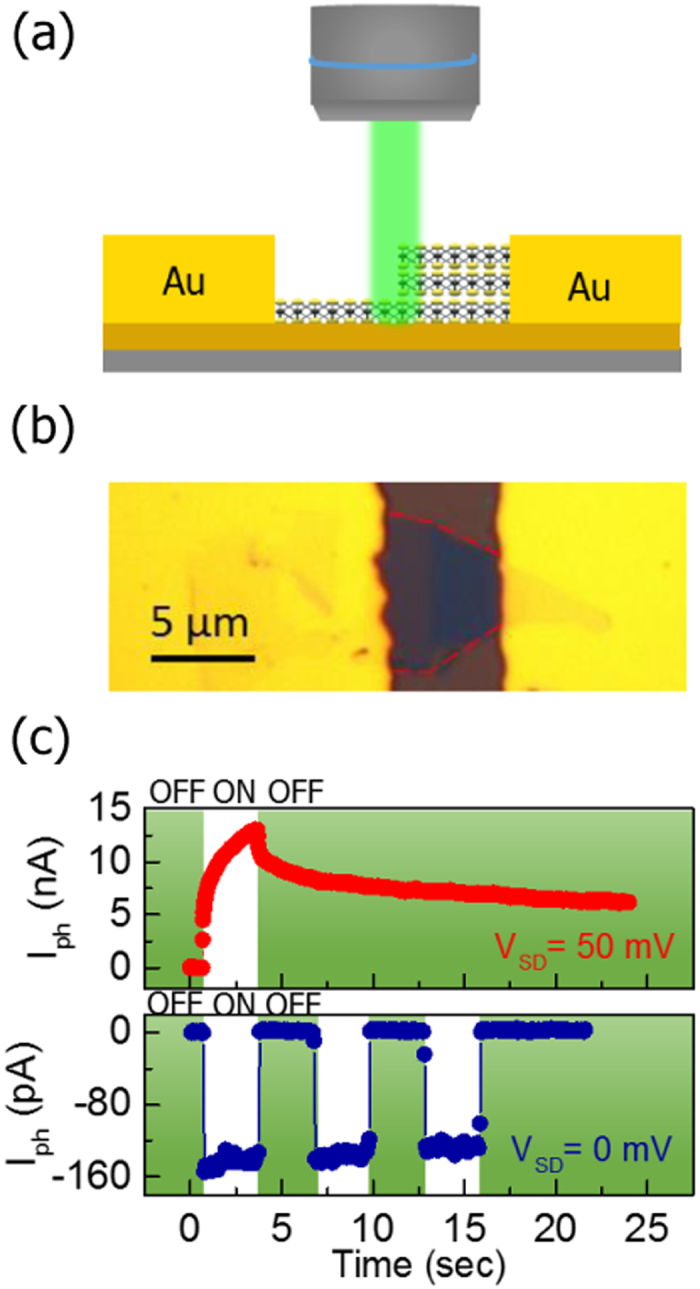
The structure and the photoresponse behaviors of the MoS_2_ junctions. (**a**) A schematic diagram of a 1L-3L MoS_2_ junction transistor with the excitation beam focused on the MoS_2_ junction. (**b**) Optical image of a MoS_2_ junction transistor. The edge of the MoS_2_ junction is outlined by red lines. (**c**) Time-resolved photoresponse behaviors of the MoS_2_ junction transistor (sample A) under *V*_*SD*_ = 50 mV (red curve) and *V*_*SD*_ = 0 mV (blue curve) at *V*_*G*_ = 60 V.

We studied the photoresponse behavior of the MoS_2_ junction devices to investigate the PV effect due to the built-in electric field. Figure 1a,b show a schematic of the device structure of MoS_2_ 1L-3L junction with Au electrodes and an optical microscopy image of a typical MoS_2_ junction device (sample A), respectively. For sample A, the total channel length is 5 μm, and the thin and thick regions of the MoS_2_ junctions are 0.7 and 1.9 nm, respectively. We performed the photocurrent measurement with a photoexcitation (532 nm, 20 kW/cm^2^) focused at the MoS_2_ junction interface at *V*_*G*_ = 60 V. Figure 1c compares the photocurrent at *V*_*SD*_ = 0 mV and *V*_*SD*_ = 50 mV, in which we readily found drastically different photoresponse between zero and small *V*_*SD*_. For *V*_*SD*_ = 50 mV, the photocurrent exhibits a PPC which is the photocurrent that persists after the photoexcitation is terminated. For a disordered system, it is common to use the stretched exponential decay to describe the PPC relaxation^30^,^31^,^32^. Here, we can analyze the photocurrent relaxation by a single stretched exponential decay^33^


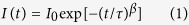


where *τ* is the decay time, and *β* is the exponent (0 < *β* < 1), yielding *τ* = 90 s for *V*_*SD*_ = 50 mV. The decay time is comparable to the previous study^30^, suggesting that the observed PPC effect in the MoS_2_ sample is originated from random localized potential fluctuation^30^,^31^,^32^. In contrast, the short-circuit photocurrent (*I*_*SC*_) at zero bias is simply due to the PV effect driven by the built-in electric field, which exhibits a fast switching behavior and returns to the dark current level rapidly after illumination is terminated. The photocurrent relaxation can be fitted by a normal exponential decay (*β* = 1 in Equation 1)^34^,^35^,^36^. The value of *τ* is extracted to be approximately 60 ms, which are smaller than that under bias (*V*_*SD*_ = 50 mV) by 3 orders of magnitude (Supporting information 3).

The drastic difference between photoresponse with and without applying *V*_*SD*_ is intriguing and can be attributed to the difference in the carrier transport mechanism^37^. Here, we mainly focus on the slow-changing photocurrent that distinguishes the two scenarios. The current in the channel can be expressed as *J*_*n*_ = *J*_*n*_(*drift*) + *J*_*n*_(*diffusion*) = *eμ*_*n*_*nE* − *eD*_*n*_*dn/dx*. The carrier density is varied temporally due to the trapping and de-trapping process in the illumination and dark condition, respectively. Because the photoconductivity (PC) is determined by the drift current, which is proportional to the carrier density, the PC is greatly affected by the presence of the charge traps. In contrast, when no bias voltage is applied, *I*_*SC*_ is induced by the built-in electric field at the junction and then driven by the diffusion current in the channel, which is proportional to the gradient of the carrier density *dn/dx*. Because *dn/dx* is related to the gradient of quasi-Fermi level *dF*_*n*_/*dx* but not the carrier density, it is much less sensitive to the trapping/de-trapping processes. Therefore, *I*_*SC*_ exhibits a much faster photoresponse and the PPC effect can be greatly suppressed when the photocurrent is dominated by the short-circuit current.

**Figure 2:**
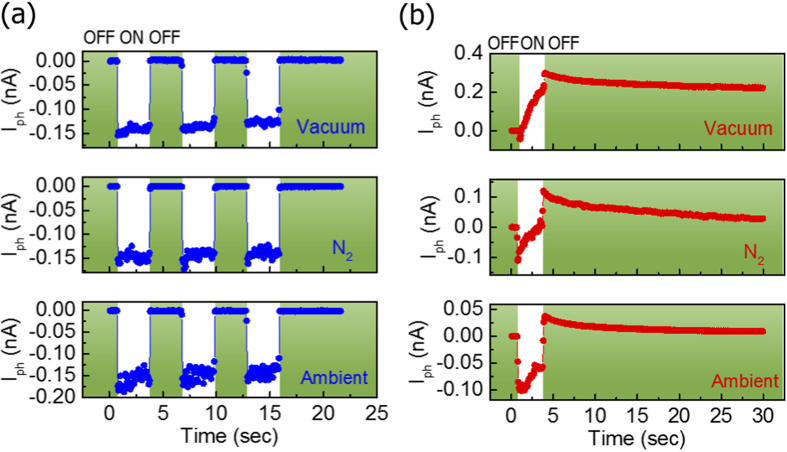
Time-resolved photoresponse of the MoS_2_ junctions. The photocurrent behavior of the MoS_2_ junction transistor (sample B) under different gaseous conditions at (**a**) *V*_*SD*_ = 0 mV and (**b**) *V*_*SD*_ = 5 mV. The short-circuit photoresponse is virtually insensitive to the variation of the gaseous conditions.

Interestingly, the short-circuit photocurrent is virtually insensitive to the variation of the gaseous conditions over a range from a vacuum of 1 × 10^−6^ Torr to ambient condition. Here, we present this unique characteristic by showing the*V*_*SD*_ dependent photocurrent (*I*_*ph*_) in different gaseous environment. Figure 2a compares *I*_*ph*_ of a 1L-3L MoS_2_ junction (sample B) at*V*_*SD*_ = 0 mV among vacuum, N_2_ (1 atm), and ambient condition, revealing that the magnitude of the *I*_*SC*_ (Δ*I*_*SC*_) is insensitive to the environment. We note that in all 5 1L-3L samples that we measured, the photoresponse behavior is comparable and therefore the data shown here is representative. Under very different pressure and chemical substances, Δ*I*_*SC*_ exhibits essentially the same value of approximately 140 pA. Moreover, the decay time (τ) values for these different conditions are also similar, as described in detail in Supporting information S3. In contrast, the photocurrent of sample B under bias critically depends on the environmental conditions. Figure 2b shows *I*_*ph*_ at*V*_*SD*_ = 5 mV under vacuum, N_2_, and ambient conditions, indicating the changes in photocurrent (Δ*I*_*PC*_) for these three conditions are 263, 133, and 41 pA, respectively. Here we note that while Δ*I*_*SC*_ is only governed by the built-in electric field, Δ*I*_*PC*_ is referred to PC that is determined by both built-in field and external bias. Moreover, the sample exhibits largerτ under vacuum (90 sec) as compared to the τ under N_2_ (16 sec) and ambient (8 sec) conditions. This dependence of *I*_*ph*_ on the chemical environment is typical for the 2D-material devices. The extracted parameters of mobility, threshold voltage, Δ*I*_*SC*_, and Δ*I*_*PC*_ are listed in Table 1.

As previously discussed, *I*_*SC*_ is driven by the diffusion current that is related to the gradient of carrier density. This gradient is associated with the condition of illumination, but is negligibly affected by the trapping process and the presence of adsorbents. Consequently, both the magnitude and the response time of *I*_*SC*_ are very insensitive to the gaseous environment, despite the pressure and the chemical composition being very different. We note that this insensitivity of the photocurrent in MoS_2_ junctions is very uncommon in 2D-material-based devices, considering the ultrahigh surface-to-volume ratio that leads to a large area being exposed to absorbents. Conversely, when bias voltage is applied, the carriers could be trapped or released during illumination and dark conditions, leading to the charging and the PPC effect, as seen in Fig. 2b.

**Figure 3:**
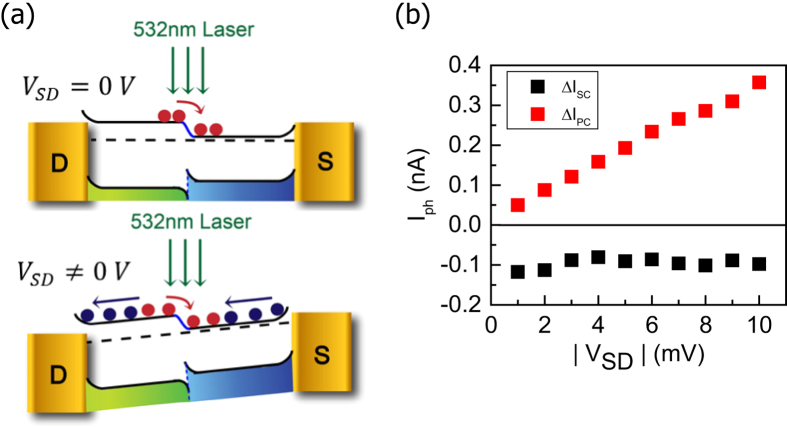
V_SD_-dependent photoresponse of the MoS_2_ junctions. (**a**) A schematic of the band structure of the MoS_2_ junction transistor and photoinduced carrier transfer at *V*_*SD*_ = 0 V and *V*_*SD*_ ≠ 0 V. (**b**) The photocurrent measurement of sample B corresponding to the photovoltaic effect (black squares) and the photoconductivity effect (red squares) as a function of *V*_*SD*_ at *V*_*G*_ = 60 V. The excitation power is 200 μW.

**Figure 4:**
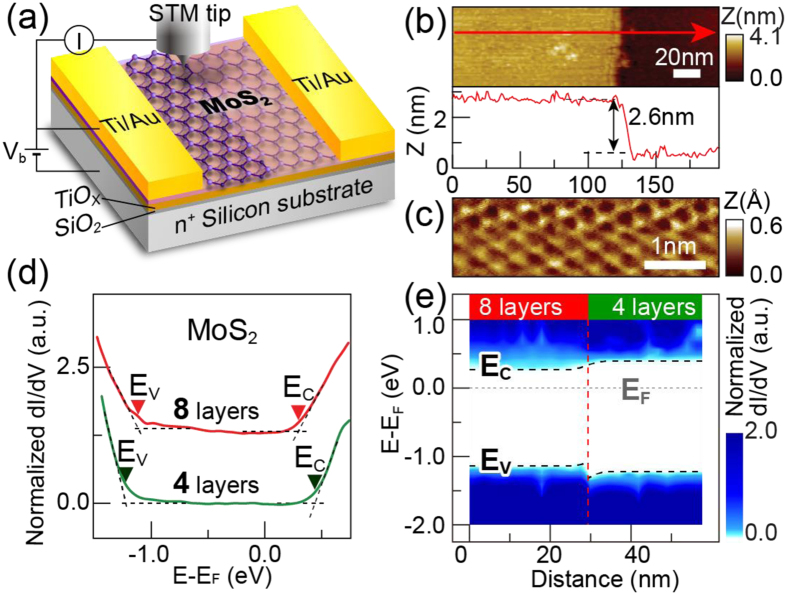
The STM/STS measurement of the MoS_2_ junctions. (**a**) A schematic of the MoS_2_ junction structure with different layer in the STM/STS measurement. (**b**) Top: STM topography image of the MoS_2_ junction (sample C). Bottom: a cross-sectional topographic profile of the MoS_2_ junction. (**c**) An STM image of sample C with atomic-scale resolution, which indicates a pristine MoS_2_ surface. (**d**) Normalized *dI/dV* curves of 4 layer (green curve) and 8 layer (red curve) MoS_2_. The profiles are offset for clarity. (**e**) Band alignment across the MoS_2_ 4- to 8-layer junction. Type-I band alignment at the MoS_2_ junction was implied.

To further investigate the observed PV effect due to the built-in electric field, we present the *V*_*SD*_ dependence of the photocurrent. Figure 3a shows a schematic diagram of energy band alignment and photoinduced carrier dynamics in the MoS_2_ heterojunction. Type-I band alignment is implied based on the STS results shown in Fig. 4d,e. At zero bias, the photocurrent is mainly driven by the built-in field under illumination (Supporting Information S4), whereas both the built-in and the external field co-exist when bias voltage is applied. It is noted that only the electron conduction is considered here because all the MoS_2_ junction samples exhibit n-type semiconducting behavior (Supporting Information S1). We considered the photocurrent under two polarity of bias (the detailed calculation of Δ*I*_*SC*_ and Δ*I*_*PC*_ are described in Supporting Information S5). The fast photoresponse of the photocurrent is negative, regardless of the polarity of bias, indicating that *I*_*SC*_ is induced by the built-in electric field. We then compared the photocurrent of sample B corresponding to the PV and the charging/discharging effect as a function of *V*_*SD*_ in small bias regime, as shown Fig. 3b. Δ*I*_*SC*_ is found to be relatively independent of *V*_*SD*_; this response is ascribed to the cancellation of the external field. In contrast, Δ*I*_*PC*_, and thus the charging/discharging effect, increases linearly with *V*_*SD*_; this response may be due to the phenomenon that the carriers are driven to the local potential minimum more efficiently with increasing bias.

Figure 4a shows a schematic of the experimental setup for the STM measurement on the MoS_2_ junction transistors. Before MoS_2_ exfoliation, we deposited a thin TiO_x_ film of 5 nm^38^, which served as electron transfer dopant on MoS_2_ flakes^39^, resulting in the reduction of MoS_2_ sheet resistance. The deposition of a TiO_x_ layer is therefore crucial for preventing of the STM tip from crashing during the measurement. Figure 4b shows a topography image of the MoS_2_ junction device (sample C) at the junction, revealing the step with two flat terraces. The apparent step height is 2.6 nm, corresponding to 4 layers of MoS_2_. Because the thinner MoS_2_ is characterized by AFM as 4 layers, the studied MoS_2_ sample is therefore a 4- to 8-layer junction. Figure 4c shows a high magnification topography image of MoS_2_ that reveals crystalline structure, indicating a pristine MoS_2_ surface.

To investigate the band alignment at the MoS_2_ junction, the STS technique was utilized to measure the normalized *dI/dV* as a function of bias, which corresponds to the local density of state (LDOS). Figure 4d shows the bias dependence of *dI/dV* curve for the two MoS_2_ terraces with different thicknesses. The onsets of the normalized *dI/dV* curves at positive and negative bias correspond to the conduction band edge (*E*_*C*_) and the valance band edge (*E*_*V*_), respectively (Supporting information S6). From the STS measurement, we deduce the following: the *E*_*C*_ and *E*_*V*_ in 8-layer MoS_2_ are 0.29 eV and −1.12 eV, respectively; the *E*_*C*_ and *E*_*V*_ in 4-layer MoS_2_ are 0.44 eV and −1.22 eV, respectively. By spatially mapping the normalized *dI/dV* curves, we plot the band alignment across the 4- to 8-layer junction, as shown in Fig. 4e. The deduced energy profile suggests that the band alignment of the MoS_2_ junction is type I, as depicted in Fig. 3a.

**Figure 5:**
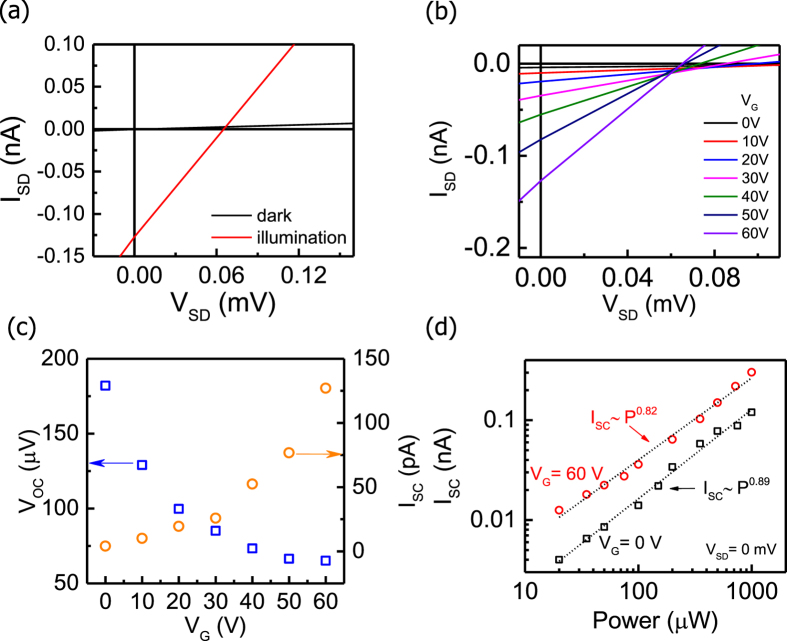
Field-effect-controlled short-circuit photocurrent of the MoS_2_ junction transistors. (**a**) Output curves of the MoS_2_ junction device (sample B) in dark (black curve) and under 532 nm excitation focused on the MoS_2_ junction (red curve) at *V*_*G*_ = 60 V. (**b**) *V*_*G*_ dependence of the output curves under laser illumination (P = 200 μW) at the MoS_2_ junction. (**c**) Analysis of *V*_*G*_ dependence of *V*_*OC*_ and *I*_*SC*_ extracted from the output curves. (**d**) Excitation power dependence of *I*_*SC*_ at zero bias at *V*_*G*_ = 0 V (black squares) and *V*_*G*_ = 60 V (red circles). The dashed lines are fitting curves of power law *I*_*SC*_ ∝ *P*^*α*^.

Finally, we present the current-voltage characteristics at different *V*_*G*_ to analyze the field effect of the photocurrent in the MoS_2_ junction devices. Figure 5a compares the *I*_*SD*_ − *V*_*SD*_ curves of sample B in dark and under illumination with a 532 nm laser and 20 kW/cm^2^ at *V*_*G*_ = 60 *V*. The *I*_*SD*_ − *V*_*SD*_ curves exhibit a linear behavior, indicating that resistor behavior, rather than the rectifying effect, dominates the transport property of the junctions. The slope of the *I*_*SD*_ − *V*_*SD*_ curves therefore approximates the conductance (*G*) of the device. Under illumination, *G* is enhanced by 40 times compared with *G* in dark; this enhancement is attributed to the generation of the photoinduced carriers. The open-circuit voltage (*V*_*OC*_) and *I*_*SC*_ can be extracted as 60 *μ*V and 0.12 nA, respectively. We note that the *I*_*SC*_ can be further enhanced by reducing the Schottky barrier height via contact engineering.

Because the Fermi level in thin materials can be effectively tuned by the external electric field, it is intriguing to study the field effect of the current-voltage characteristics. Indeed, we observed a *V*_*G*_ dependence of the *I*_*SD*_ − *V*_*SD*_ curves under illumination, as shown in Fig. 5b, in which *G* increases with increasing *V*_*G*_. To understand this field effect, we plot *V*_*OC*_ and *I*_*SC*_ as a function of *V*_*G*_, as shown in Fig. 5c. *I*_*SC*_ is found to increase with increasing *V*_*G*_, similar to the *I*_*SD*_ − *V*_*G*_ curve (see Figure S1 of SI 1). This similarity is reasonable because *I*_*SC*_ depends on the collection probability of the photoinduced carriers, which is correlated to the diffusion current and *G*. Moreover, as *V*_*G*_ increases, the contact resistance may decrease due to Schottky barrier thinning^40^, leading to higher *I*_*SC*_. Conversely, the response of *V*_*OC*_ decreasing with increasing *V*_*G*_ can be attributed to the reduction of built-in electric field in the MoS_2_ junction. Because monolayer MoS_2_ is subjected to a stronger field effect compared with the few-layer MoS_2_ due to the different density of states, the rising of Fermi level in monolayer MoS_2_ is greater than that in few-layer MoS_2_ as *V*_*G*_ increases, resulting in the reduction of the built-in electric field (see Fig. 3a) and thus *V*_*OC*_.

We further present the excitation power dependence of *I*_*SC*_ at zero bias voltage (Fig. 5d) to examine the mechanism of the photocurrent generation^41^. We observe that *I*_*SC*_ follows a power law *I*_*SC*_ ∝ *P*^*α*^, and the exponent α can be been extracted as 0.89 and 0.82 for *V*_*G*_ = 0 and 60 V, respectively. For the PC and the PV process, the photoinduced carrier density is directly proportional to the rate of absorbed photons; therefore, α = 1^42^. However, because the PC is excluded here (*V*_*SD*_ = 0), the value of α therefore suggests that the PV is the dominant mechanism in the measured photocurrent. The deviation of the extracted α from unity may be attributed to electron-hole recombination at the MoS_2_ junction and/or nonradiative recombination centers^43^,^44^.

In conclusion, we demonstrated a unique environment-insensitive and gate-controllable short-circuit photocurrent in a MoS_2_ junction with differences in the number of layer. The environmental insensitivity of the short-circuit photocurrent can be attributed to the characteristic of the diffusion current. Conversely, the photocurrent with bias exhibits the typical PPC that greatly depends on the amount of the extrinsic adsorbents. The STM/STS measurement confirms the quality of the MoS_2_ junction samples and suggests the type-I band alignment of the junction. In addition to the effect of source-drain bias, the MoS_2_ junction devices exhibit strong back-gate voltage dependence, indicating the feasibility to control the photocurrent via field effect. The environment-insensitive photocurrent therefore shows an alternative method to design the device structure for TMDC-based electronic and optoelectronic applications.

## Methods

### Sample preparation

The sample with the 1L-3L MoS_2_ junction was produced via mechanical exfoliation of MoS_2_ layers from the bulk MoS_2_ (SPI supplies) onto SiO_2_ (300 nm)/Si substrates. Next, a resist-free technique with a shadow mask (TEM grids) was utilized to deposit electrical contacts. The advantage of the resist-free technique is the lack of resist residue on the MoS_2_ surface resulting from the device fabrication process. We deposited Au (50 nm) as the electrical contacts using an electron-beam evaporator at a base pressure of 1.0 × 10^−7^ Torr. All of our MoS_2_ junction devices were measured in a cryostat (Janis Research Company, ST-500) under vacuum condition of 1.0 × 10^−6^ Torr. We performed DC electrical measurement using a Keithley 237 sourcemeter and applied the back-gate voltage using a Keithley 2400 sourcemeter. We employed solid-state CW laser (Nd:YAG, 532 nm) as the light source in the Raman spectroscopy and photoresponse measurements. The incident light beam was focused by an objective (100×, NA 0.6) with a spot size of ~0.9 μm.

## Additional Information

**How to cite this article:** Shih, F.-Y. *et al*. Environment-insensitive and gate-controllable photocurrent enabled by bandgap engineering of MoS_2_ junctions. *Sci. Rep.* **7**, 44768; doi: 10.1038/srep44768 (2017).

**Publisher's note:** Springer Nature remains neutral with regard to jurisdictional claims in published maps and institutional affiliations.

## Supplementary Material

Supplementary Information

## Figures and Tables

**Table 1:** The characteristics of the MoS_2_ junction (sample B) under different gaseous conditions.

	Vacuum	N_2_	Ambient
Mobility	0.5 *cm*^2^/*Vs*	0.14 *cm*^2^/*Vs*	0.09 *cm*^2^/*Vs*
Threshold voltage	5.5 V	20 V	25.5 V
**Δ*****I***_***SC***_			
V_SD_ = 0 mV	−136 pA	−143 pA	−149 pA
**Δ*****I***_***PC***_			
V_SD_ = 5 mV	263 pA	133 pA	41 pA
