# Supplementary material for: Environment-insensitive and gate-controllable photocurrent enabled by bandgap engineering of MoS2 junctions
Source: Sci Rep. 2017 Mar 21;7:44768. doi: 10.1038/srep44768 (PMC5359557; doi:10.1038/srep44768)
Supplement: Supplementary Information [file srep44768-s1.pdf]

Supporting Information

Environment-insensitive and gate-controllable photocurrent enabled by bandgap engineering of MoS_2 junctions

Fu-Yu Shih[1,2], Yueh-Chun Wu[2,§], Yi-Siang Shih[1], Ming-Chiuan Shih[3], Tsuei-Shin Wu[1,2], Po-Hsun Ho[4], Chun-Wei Chen[4], Yang-Fang Chen[1], Ya-Ping Chiu[1,5], and Wei-Hua Wang[2*]

[1]Department of Physics, National Taiwan University, Taipei 106, Taiwan

[2]Institute of Atomic and Molecular Sciences, Academia Sinica, Taipei 106, Taiwan

[3]Department of Physics, National Sun Yat-sen University, Kaohsiung, Taiwan

[4]Department of Materials Science and Engineering, National Taiwan University, Taipei 106, Taiwan

[5]Institute of Physics, Academia Sinica, Taipei 115, Taiwan

[§]Current address: Department of Physics, University of Massachusetts, Amherst, Massachusetts 01003, United States

[*]Corresponding Author. (W.-H. Wang) Tel: +886-2-2366-8208, Fax: +886-2-2362-0200;

E-mail: wwang@sinica.edu.tw

S1. Device fabrication and photocurrent measurement

The sample with the 1L-3L MoS_2 junction was produced via mechanical exfoliation of MoS_2 layers from the bulk MoS_2 (SPI supplies) onto SiO_2 (300 nm)/Si substrates. Next, a resist-free technique with a shadow mask (TEM grids) was utilized to deposit electrical contacts. The advantage of the resist-free technique is the lack of resist residue on the MoS_2 surface resulting from the device fabrication process. We deposited Au (50 nm) as the electrical contacts using an electron-beam evaporator at a base pressure of 1.0×10^{-7} Torr. All of our MoS_2 junction devices were measured in a cryostat (Janis Research Company, ST-500) under vacuum condition of 1.0×10^{-6} Torr. We performed DC electrical measurement using a Keithley 237 sourcemeter and applied the back-gate voltage using a Keithley 2400 sourcemeter. We employed solid-state CW laser (Nd:YAG, 532 nm) as the light source in the Raman spectroscopy and photoresponse measurements. The incident light beam was focused by an objective (100×, NA 0.6) with a spot size of ~ 0.9 μm.

Figure S1 compares the $I_{SD} - V_G$ curves of a 1L-3L MoS_2 junction device (sample B of the main text) under vacuum, N_2, and ambient conditions. The MoS_2 junction device exhibits typical n-type semiconducting behavior. The sample exhibits higher mobility under vacuum (0.5 $\mathrm{cm}^2/\mathrm{Vs}$) compared to the mobility under N_2 (0.14 $\mathrm{cm}^2/\mathrm{Vs}$) and ambient (0.09 $\mathrm{cm}^2/\mathrm{Vs}$) conditions. This difference can be understood because the carrier scattering is higher when additional adsorbents are introduced in the environment with abundant molecules [1]. The on/off ratio is approximately 10^4. The threshold for N_2 and ambient conditions is higher than that under vacuum, suggesting a p-type doping effect is introduced during the adsorption [2, 3].

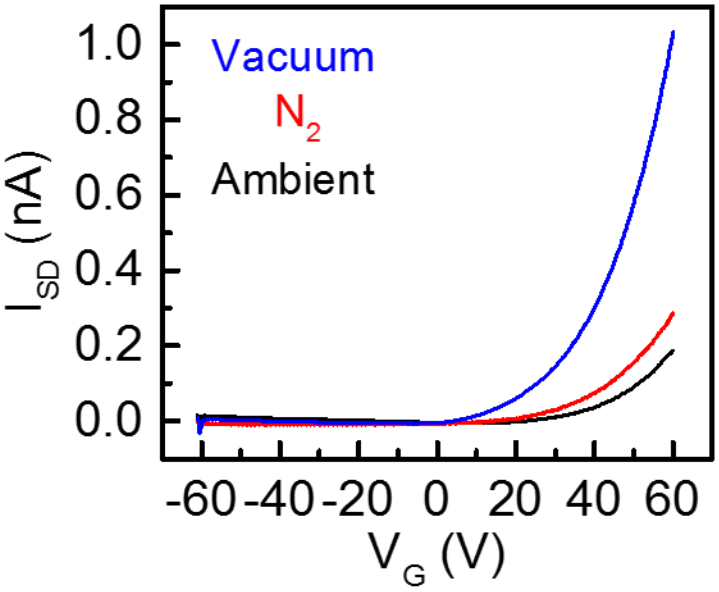

Figure S1. The transfer curves of 1L-3L MoS_2 junction device under vacuum, N_2, and ambient conditions.

S2 Identification of the number of MoS_2 layers

The layer number of the MoS_2 flakes was identified using optical microscopy, Raman spectroscopy, and atomic force microscopy (AFM) measurements. Figure S2a presents an optical image of MoS_2 flake on 300-nm SiO_2/Si substrate involving monolayer and trilayer MoS_2 (sample A of the main text). The Raman spectra (Figure S2b) reveals two characteristic peaks 388.1 (386.6) cm^{-1} and 407 (409.1) cm^{-1} of the monolayer (trilayer) MoS_2 flake, which correspond to the E^1_{2g} and A_{1g} resonance modes, respectively. The difference between the two peaks can be used to identify the thickness of the MoS_2, particularly for a number of layers less than 3 [4]. For this MoS_2 junction sample, the difference is equal to 18.9 cm^{-1} for monolayer MoS_2 and 22.5 cm^{-1} for trilayer MoS_2, in agreement with previous reports [4]. We performed AFM measurement in a region denoted by a red rectangle shown in Figure S2a. The result is presented in Figure S2c, which shows that the step height of the thinner area of the MoS_2 junction was approximately 0.7 nm, which corresponds to one atomic layer. The step height of the thicker area of the MoS_2 junction was approximately 1.9 nm, which corresponds to trilayer MoS_2.

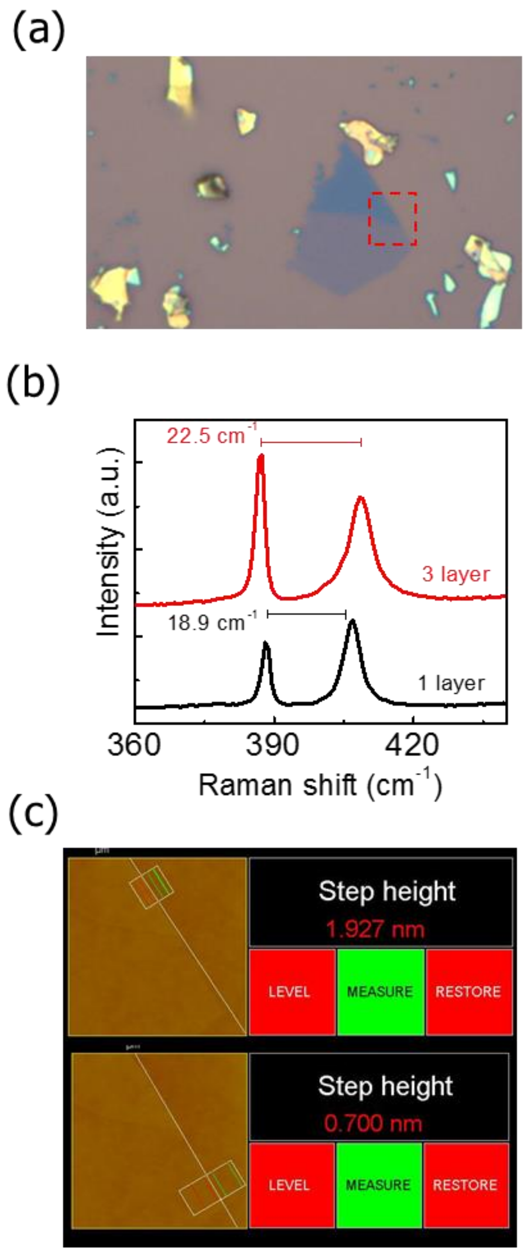

Figure S2. (a) Optical microscope image and (b) Raman spectra and (c) AFM image of 1L-3L MoS_2 junction (Sample A).

S3 Estimation of the decay time under different gaseous conditions

We measured the time-resolved photocurrent by using the built-in sweep function of the Keithley 237 sourcemeter with the setting-reading cycle of 18 ms. Figure S3a shows the time-resolved photocurrent (black squares) at zero bias voltage under ambient, N_2, and vacuum condition, respectively along with the fitting curves (red lines) based on normal exponential decay, $I(t) = I_0 exp - (t/\tau)$. The values of τ under ambient, N_2, and vacuum condition were extracted as 61.8 ms, 63.8 ms, and 62.1 ms, respectively. The value of τ is found to be approximately the same under different environmental conditions. Figure S3b shows time-resolved photocurrent at $V_{SD} = 5\,mV$ under ambient, N_2 and vacuum conditions along with the fitting curves (green lines) based on stretched exponential decay, $I(t) = I_0 exp[-(t/\tau)^\beta]$. The extracted τ is found to be dependent on the environment; this result can be attributed to the random localized potential fluctuations in MoS_2 [5].

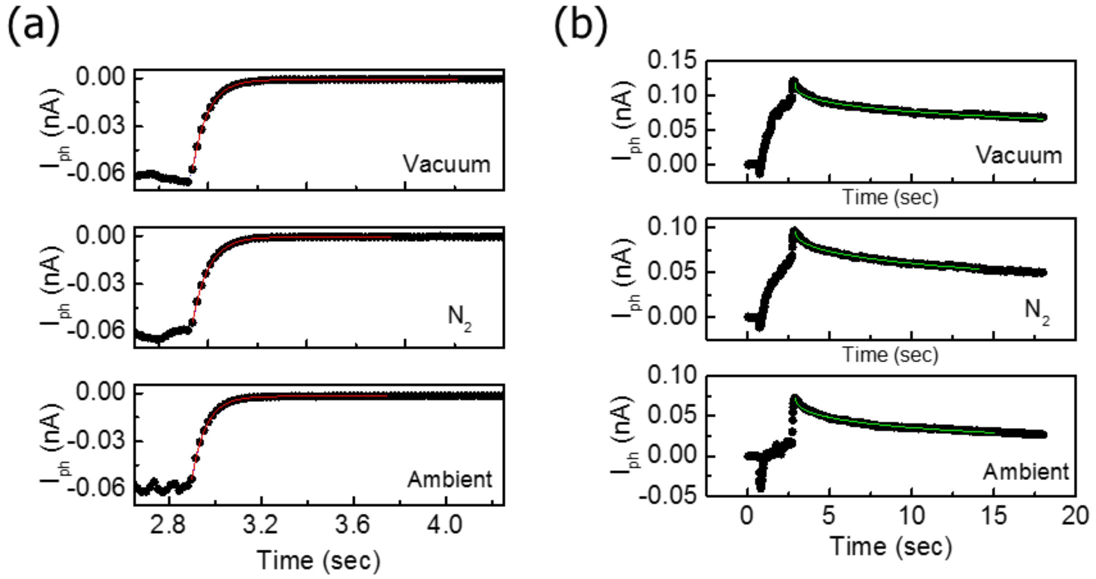

Figure S3. Time-resolved photocurrent measurement at (a) zero bias and (b) $V_{SD} = 5\,mV$ under different gaseous conditions. The red and green curves represent the fitting curves based on normal exponential decay and stretched exponential decay, respectively.

	Vacuum	N_2	Ambient
decay time (τ) V_{SD} = 0 mV	62.1 ms	63.8 ms	61.8 ms
decay time (τ) V_{SD} = 5 mV	64 s	37.4 s	15.2 s

Table S1. The decay time of the MoS_2 junction (sample B) under different gaseous conditions.

S4 Zero-bias time-resolved photoresponse in a uniform MoS_2 transistor

The observed zero-bias photocurrent in the MoS_2 junction device is due to the band offset resulting from the different thicknesses of MoS_2. To verify this characteristic, we present the zero-bias photocurrent measurement in a uniform MoS_2 device, which shows no photoresponse when illuminated at zero bias (Figure S4). This lack of photoresponse is in contrast to the observed photoresponse measured in the MoS_2 junction device, as shown in Figures 1c and 2a in the main text.

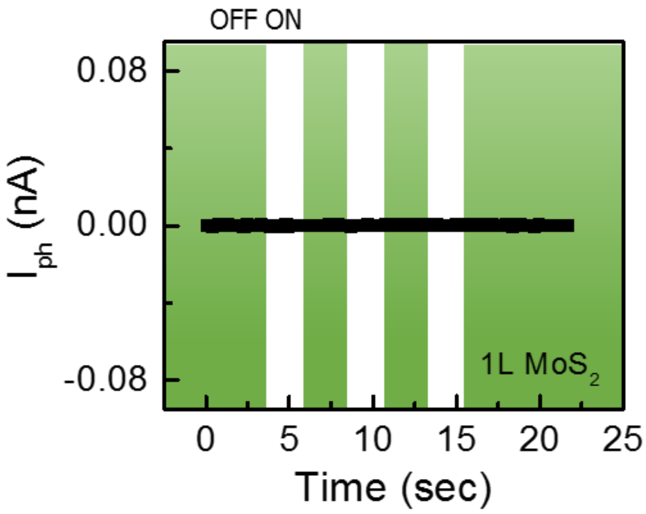

Figure S4. Time-resolved photoresponse of uniform 1L MoS_2 at zero-bias voltage.

S5 Analysis of V_{SD} dependence of the photocurrent due to the PV and the PC effect

Figures S5a and S5b show the temporal photoresponse of the 1L-3L MoS_2 junction device under small bias ($V_{SD} = \pm 2$ mV). The direction of the photocurrent with slow response is found to depend on the direction of the external electric field. In contrast, the direction of the photocurrent with quick response is observed to retain a specific direction, suggesting that the photocurrent with quick response is mainly caused by the junction-induced built-in electric field.

To distinguish the photocurrent corresponding to the PV effect versus the PC effect, we define I_{SC} and I_{PC} as:

$I_{SC} = \left(I_{V_{SD}=x\,mV} + I_{V_{SD}=-x\,mV}\right)/2$ Eqn. S1

$I_{PC} = \left(\left|I_{PC,V_{SD}=x\,mV}\right| + \left|I_{PC,V_{SD}=-x\,mV}\right|\right)/2$ Eqn. S2

where $I_{V_{SD}=x\,mV}$ is the change of photocurrent with quick response at $V_{SD} = x\,mV$, and $I_{PC,V_{SD}=x\,mV}$ is the change of photocurrent with slow response at $V_{SD} = x\,mV$. The photocurrent caused by external bias with quick response is cancelled by summing up the contributions from the two polarities of the bias voltages. Therefore, I_{SC} represents the photocurrent resulting from the built-in electric field. Alternatively, we take the average of the absolute value of the photocurrent with slow response under the two polarities of bias to represent the photocurrent due to the PC effect.

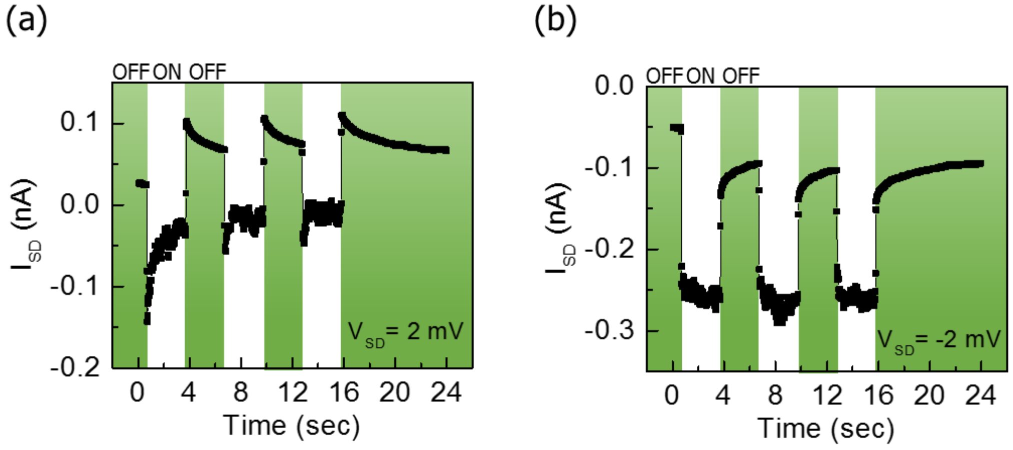

Figure S5. Time-resolved photocurrent of 1L-3L MoS_2 junction under small bias ($V_{SD} = \pm 2$ mV).

S6 STM/STS characterization

In STS measurements, the STM probe tip, vacuum and MoS_2 form a metal-insulator-semiconductor (MIS) tunneling junction. When we apply zero sample bias on the sample so that there is no tunneling current between tip and MoS_2, the Fermi-level of the tip is equal to the Fermi-level of the MoS_2 under thermal equilibrium. Therefore, the zero sample bias can be defined as the Fermi-level of the sample. In contrast to the zero sample bias condition, when we apply a certain sample bias on the sample, the tunneling current flows between the probe tip and MoS_2. As a result, the onsets in the normalized dI/dV curves indicate the current onsets from the valance band edge (E_V) and the conduction band edge (E_C) in STM measurements.

References

1. Qiu, H., et al., *Electrical characterization of back-gated bi-layer MoS2 field-effect transistors and the effect of ambient on their performances.* Applied Physics Letters, 2012. **100**(12): p. 123104.
2. Tongay, S., et al., *Broad-range modulation of light emission in two-dimensional semiconductors by molecular physisorption gating.* Nano letters, 2013. **13**(6): p. 2831-2836.
3. Yue, Q., et al., *Adsorption of gas molecules on monolayer MoS2 and effect of applied electric field.* Nanoscale research letters, 2013. **8**(1): p. 1-7.
4. Li, H., et al., *From bulk to monolayer MoS2: evolution of Raman scattering.* Advanced Functional Materials, 2012. **22**(7): p. 1385-1390.
5. Wu, Y.-C., et al., *Extrinsic Origin of Persistent Photoconductivity in Monolayer MoS2 Field Effect Transistors.* arXiv preprint arXiv:1505.05234, 2015.
